# Evaluating Compliance with Institutional Preoperative Testing Guidelines for Minimal-Risk Patients Undergoing Elective Surgery

**DOI:** 10.1155/2013/835426

**Published:** 2013-07-07

**Authors:** Arunotai Siriussawakul, Akarin Nimmannit, Sirirat Rattana-arpa, Siritda Chatrattanakulchai, Puttachard Saengtawan, Aungsumat Wangdee

**Affiliations:** ^1^Department of Anesthesiology, Faculty of Medicine, Siriraj Hospital, Mahidol University, Bangkok 10700, Thailand; ^2^Office for Research and Development, Faculty of Medicine, Siriraj Hospital, Mahidol University, Bangkok 10700, Thailand

## Abstract

*Background*. Few investigations preoperatively are important for low-risk patients. This study was designed to determine the level of compliance with preoperative investigation guidelines for ASA I patients undergoing elective surgery. Secondary objectives included the following: to identify common inappropriate investigations, to evaluate the impact of abnormal testing on patient management, to determine factors affecting noncompliant tests, and to estimate unnecessary expenditure. *Methods*. This retrospective study was conducted on adult patients over a one-year period. The institute's guidelines recommend tests according to the patients' age groups: a complete blood count (CBC) for those patients aged 18–45; CBC, chest radiograph (CXR) and electrocardiography (ECG) for those aged 46–60; and CBC, CXR, ECG, electrolytes, blood glucose, blood urea nitrogen (BUN), and creatinine (Cr) for patients aged 61–65. *Results*. The medical records of 1,496 patients were reviewed. Compliant testing was found in only 12.1% (95% CI, 10.5–13.9). BUN and Cr testings were the most frequently overprescribed tests. Overinvestigations tended to be performed on major surgery and younger patients. Overall, overinvestigation incurred an estimated cost of US 200,000 dollars during the study period. *Conclusions*. The need to utilize the institution's preoperative guidelines should be emphasized in order to decrease unnecessary testing and the consequential financial burden.

## 1. Introduction

 The fundamental purposes of preoperative investigations are to obtain information regarding a patient's fitness for anesthesia and surgery and to assess the intraoperative risks [1–4]. Preoperative investigations were found to be beneficial and cost-effective when they had been correlated with the patients' histories and physical examinations. Obtaining the results of investigations of symptomatic patients can help clinicians to confirm diagnoses, assess the severity and progression of diseases, and predict the prognoses [5]. In contrast, performing preoperative investigations in asymptomatic patients or healthy patients (The American Society of Anesthesiologists Physical Status, ASA I) may lead to many disadvantages. Firstly, the ability of preoperative investigations to predict adverse postoperative outcomes is weak [6], secondly, the tests have a low impact on clinical management, and last but not least, the tests incur a huge and unnecessary expenditure [7].

 The application of the institute's guidelines should decrease the number of laboratory tests and consequential costs with no untoward events, especially when applied to low-risk patients [3, 8]. Our hospital introduced the Siriraj Preoperative Investigation Guidelines in 2008. The guidelines are based on clinical and cost-effectiveness considerations similar to those used by other institutional guidelines [2, 3]. Few investigations prior to surgery are considered important for low-risk patients. Unfortunately, the application of the guidelines in clinical practice demonstrated a high failure rate in many reports. The proportion of patients who underwent at least one nonindicated test according to their institutional guidelines ranged from 60 to 90% [1, 9]. The types of common noncompliant tests were chest radiograph (CXR), electrocardiography (ECG), liver function test (LFT), and coagulogram [10, 11].

 The objectives of this study were, firstly, to determine the proportion of low risk patients (ASA I) who underwent compliant testing. The study's secondary objectives included the following: to identify common inappropriate investigations, to evaluate the impact of abnormal testing on management, to determine factors affecting noncompliant tests, and to estimate the level of unnecessary expenditure. 

## 2. Materials and Methods

 After Institution Review Board approval, a retrospective study involving patients having undergone operations between June 1, 2010, and May 31, 2011, was performed. Data sources were Siriraj Hospital's electronic medical records, and department and billing records. 

 The medical records utilized in this study comprised in-patient or ambulatory ASA I patients aged 18–65 years who had undergone elective surgery in the specialties of general surgery, gynecology, ophthalmology, orthopedics, or otolaryngology. All patients with uncertain ASA physical status were excluded from the study. Patients who had incomplete or missing data were also excluded. The institution's preoperative investigation guidelines recommend routine tests according to patients' age groups: a complete blood count (CBC) for those patients aged 18–45; CBC, CXR, and ECG for patients aged 46–60; and CBC, CXR, ECG, electrolytes, blood sugar (BS), blood urea nitrogen (BUN), and creatinine (Cr) for patients aged 61–65. Most routine orderings were prescribed by surgical residents or attending staff.

 A compliant laboratory test was defined as one that followed the institution's guidelines, whereas a noncompliant test was defined as one involving over- or under-investigation. Normal test results were based on the provided laboratory reference range of normal laboratory values. 

 Data on the following was collected: demographic data; surgical procedures; the extensiveness of surgery, which was classified as either major surgery (major thoracic or major abdominal surgery, major vascular surgery, predicted operative time ≥3 h, and predicted blood loss ≥1 L) or minor surgery; the date of preoperative testing; the types of preoperative testing (CBC, CXR, ECG, BUN, Cr, electrolyte, coagulogram and liver function test); and the test results. Regarding noncompliant testing, results of abnormal tests were explored; the line of patient management was identified (cancellation, postponement or proceeding, to surgery), and the costs of over-investigation were estimated. 

## 3. Statistics

 The primary objective of the study was to estimate the proportion of compliant tests. The sample size calculation was based on an expected incidence of compliant tests of 30% [1, 9] of the patients undergoing surgery; in order to obtain a 95% confidence interval (CI) of 5%, a sample of at least 1,291 subjects was required. However, the sample size was inflated by 15% due to incomplete information in records; therefore, 1,500 subjects were needed in this study. 

 The medical records of all ASA I patients were identified. Subsequently, stratified random sampling using the program from http://www.randomizer.org/ was performed to select 125 charts from each calendar month during the 12-month study period. Demographic variables, which were age, specialties, the extensiveness of surgery, and the period of the academic year, were then assigned to categorical data. Descriptive statistics were calculated to describe the clinical characteristics, the incidence of compliant testing and laboratory results. As for possible factors related to noncompliant testing, four factors obtained from the literature [6, 8, 12] and expert opinion were selected. Univariate differences between the compliant testing group and the noncompliant testing group were tested using the chi square test or Fisher's exact test. Variables with *P* < 0.2 from the univariate analysis were entered into a multiple logistic regression model. Data was presented as number (percent) or 95% CI, as appropriate. *P* < 0.05 (2-sided) was considered to indicate statistically significant differences. Adjusted odds ratio and 95% CI were reported to consider the strength of association between possible factors associated with noncompliant testing. Statistical analysis was conducted using the software program, SPSS version 18, SPSS Inc., Chicago, IL, USA.

## 4. Results

 A review was conducted of the medical records of 1,496 patients who had undergone elective orthopedics (26.3%), gynecology (24.9%), ophthalmology (6.2%), otolaryngology (16.6%), and general surgery (26.0%). There were 953 patients (63.7%) aged 18–45, 492 patients (32.9%) aged 46–60, and 51 patients (3.4%) aged 61–65. Most patients (89.4%) underwent major surgery (Table 1).

 Compliant preoperative testing was performed on only 12.1% (95% CI, 10.5–13.9) of the patients. Blood urea nitrogen and creatinine testing were the most common unnecessary tests (*n* = 975), followed by electrolytes (*n* = 895) and chest radiography (*n* = 763). Overall, the cost of over-investigation was estimated to be around $18,000, based on billed institutional charges. The annual excess expenditure incurred for around 15,000 ASA I patients who had undergone elective surgery was extrapolated to be more than $200,000. 

 Focusing on the data for the overprescribed tests, the proportion of abnormal tests, compared to reference range, varied (Table 2). These abnormalities were not distinctive in apparently healthy individuals and did not convince clinicians to give specific treatment because of the test results. Furthermore, all patients who underwent the operation followed surgical schedules. 

 Four factors which were probably associated with the noncompliant tests, namely, the patients' age, specialty, the extensiveness of the operations, and the periods of training programs, were included in the univariate analysis and multiple logistic regression. Three factors were found to be independently related to the noncompliant tests: young patients, patients undergoing otolaryngology or general surgery, and patients undergoing major operations. The period of training, which begins in June and ends in May of the following calendar year, had no significant impact on the compliance levels of preoperative test prescribing (*P* = 0.438) (Table 3).

## 5. Discussion

 The main finding of this study is that among ASA I patients, the proportion of patients who underwent compliant testing was low. Abnormal test results rarely led to changes in management. All patients undergoing the operation followed the surgical schedules. The most common overprescribed tests in our institution were BUN, Cr, electrolytes, and CXR. Over-investigations tended to be performed on younger patients, patients undergoing otolaryngology or general surgery, and patients undergoing major surgery. Noncompliant testing incurred a huge level of unnecessary expenditure. 

 Although the preoperative investigations for ASA I patients hava a low yield and contribute to the soaring costs of Medicare, the level of compliance with the guidelines in our institution and others [10] is low. These results were not different from previous studies. The authors conducted a retrospective review, and they found that 50–90% of patients underwent at least one nonindicated preoperative test [6, 9]. Regarding a qualitative study, the reasons why clinicians were reluctant to change their behaviors and continued to order batteries of tests were that they were concerned about surgery delays, cancellations, or medicolegal worries. Some of them lacked awareness of the guidelines or believed that other physicians required the tests to be done [13]. Surgeons, junior staff, and resident staff were the sources of duplicate tests or over-investigation. Reports demonstrated that the number of unnecessary tests climbed when they were informed that their performance in ordering investigations would be monitored or when they were concerned about test omissions and cancellations [14, 15]. Our data also demonstrated that noncompliant rates were high when the preoperative investigations were ordered by residents trained in the programs or surgical staff. 

 Successful implementation of the guidelines needs collaboration between members of a multidisciplinary team. Strategies to increase the compliance rate should be targeted at three levels, that is, the organizational level, the individual level, and the system level [16–18]. The organizational level has sole authority for writing a policy and procedural framework, and it is responsible for navigating that framework through the approval processes. Clear, consensual, and evidence-based guidelines must be developed for and promoted to clinicians. Our guidelines recommend more types of preoperative tests than other agencies (National Institute of Health and Clinical Excellence (NICE) [19], Canadian Anesthesiologists' Society (CAS) [20], or Guidelines from French Society of Anesthesia and Intensive Care [21]) because most Thai doctors are concerned about the high prevalence of certain diseases, such as tuberculosis [22], anemia, or malnutrition, which are common in our population. As a result, CBC and CXR were recommended for most patients even though they did not have clinical abnormalities. Some supplementary interventions should be executed, such as strict enforcement, the development of a sense of ownership and belief in the necessity of the guidelines, and the continual auditing of compliant levels and individualized feedback when guideline deviations are found [14]. A multidisciplinary team should also keep the guidelines current through periodic review.

We hypothesized that the rate of compliance with the guideline would increase from the beginning to the end of the one-year training period. However, our findings demonstrated that the compliance rates did not correlate with the timeline. The result might imply that our curriculum for preoperative preparation for resident staff is invalid. Since education intervention has long been shown to be effective in altering clinicians' behavior [23], the integration of up-to-date, evidence-based guidelines into the curricula is important to ensure sustainability in practice. Academic leaders from diverse universities and colleges should change the fragmented, outdated, and static curricula and try to build a comprehensive framework that better connects education with the health care system.

 Our study has some limitations. Firstly, this was a retrospective study. In addition, we did not have any information relating to the reasons why clinicians ordered unnecessary tests. Moreover, uncertain ASA classification may have caused misunderstandings; for example, healthy patients who were diagnosed with an early stage of cancer needed a metastatic survey. Since we did not apply the definition of ASA homogeneously, overinvestigations were identified in patients' diagnosed with breast cancer or gynecologic cancer.

 In conclusion, guidelines by themselves cannot increase compliance rates of routine preoperative investigation. Further studies should identify the problem related to adoption and dissemination guidelines in order to reduce the significant financial burden. 

## Figures and Tables

**Table 1:** Demographic data.

Variables	*n* (%)
Age (years)	
18–45	953 (63.7)
46–60	492 (32.9)
61–65	51 (3.4)
Specialties	
Orthopedics	393 (26.3)
Gynecology	373 (24.9)
Ophthalmology	93 (6.2)
Otolaryngology	249 (16.6)
General surgery	388 (26.0)
Extensiveness of surgery	
Major	1337 (89.4)
Minor	159 (10.6)
Period	
June 2010–Aug 2010	374 (25)
Sep 2010–Nov 2010	373 (25)
Dec 2010–Feb 2011	375 (25)
Mar 2011–May 2011	374 (25)

**Table 2:** Results of noncompliant testing.

Tests	Number of noncompliance tests	Number of abnormal findings (%*)	Reference range	Range of abnormality (<lower limit)	Range of abnormality (>upper limit)
BUN (mg/dL)	976	80 (8.0)	7–20	6–6.9	20.1–31.1
Cr (mg/dL)	976	44 (4.5)	0.5–1.5	0.2–0.4	—
Electrolyte					
Na (mmol/L)	897	45 (5.0)	135–145	130–134	146–149
K (mmol/L)	897	55 (6.1)	3.5–5.0	3–3.4	5.1–5.2
Cl (mmol/L)	897	106 (11.8)	98–107	93–97	108–118
HCO_3_ (mmol/L)	897	112 (12.4)	22–29	17–21	30–34
BS (mg/dL)	379	76 (20.5)	74–100	68–72	101–232
Albumin (g/dL)	115	5 (4.3)	3.5–5.5	—	3–3.4
PT (seconds)	60	4 (6.6)	10.5–13.5	10.2–10.4	14-15
APTT (seconds)	60	12 (20)	24–32	16.4–23.9	—

BUN: blood urea nitrogen; Cr: creatinine; Na: sodium; K: potassium; Cl: chloride; BS: blood glucose; PT: prothrombin time; APTT: activated thromboplastin time; %*: among patients with specific tests.

**Table 3:** Factor-associated noncompliant tests.

Factors	Number of cases		Adjusted OR (95% CI)	*P* value
	Compliance (%)	Noncompliance (%)		
Age (years)				
18–45	110 (11.5)	843 (88.5)	3.83 (2.07–7.09)	<0.001
46–60	55 (11.2)	437 (88.8)	3.97 (2.08–7.58)	
61–65	17 (33.3)	34 (66.7)	1	
Specialty				
General surgery	17 (4.4)	371 (95.6)	9.73 (5.71–16.58)	<0.001
Orthopedics	29 (7.4)	364 (92.6)	5.60 (3.61–8.66)	
Ophthalmology	10 (10.8)	83 (89.2)	3.70 (1.85–7.39)	
Otolaryngology	11 (4.4)	238 (95.6)	9.64 (5.07–18.35)	
Gynecology	115 (30.8)	258 (69.2)	1	
Extensiveness of surgery				
Major	173 (12.9)	1164 (87.1)	2.47 (1.24–4.95)	0.008
Minor	9 (5.7)	150 (94.3)	1	
Period				
June 10–Aug 10	52 (13.9)	322 (86.1)	0.91 (0.60–1.39)	0.438
Sep 10–Nov 10	44 (11.8)	329 (88.2)	1.10 (0.71–1.70)	
Dec 10–Feb 11	38 (10.1)	337 (89.9)	1.31 (0.83–2.05)	
Mar 11–May 11	48 (12.8)	326 (87.2)	1	
